# Recent advances in metabolic and bariatric surgery

**DOI:** 10.12688/f1000research.7240.1

**Published:** 2016-05-24

**Authors:** Vance L. Albaugh, C. Robb Flynn, Robyn A. Tamboli, Naji N. Abumrad

**Affiliations:** 1 Department of Surgery, Vanderbilt University Medical Center, Nashville, Tennessee, 37232, USA

**Keywords:** Metabolic surgery, Bariatric surgery, Morbid obesity, adjustable gastric banding, vertical sleeve gastrectomy, Roux-en-Y gastric bypass, biliopancreatic diversion, ileal interposition, bile diversion

## Abstract

Obesity and its associated medical conditions continue to increase and add significant burden to patients, as well as health-care systems, worldwide. Bariatric surgery is the most effective treatment for severe obesity and its comorbidities, and resolution of diabetes is weight loss-independent in the case of some operations. Although these weight-independent effects are frequently described clinically, the mechanisms behind them are not well understood and remain an intense area of focus in the growing field of metabolic and bariatric surgery. Perceptions of the mechanisms responsible for the beneficial metabolic effects of metabolic/bariatric operations have shifted from being mostly restrictive and malabsorption over the last 10 to 15 years to being more neuro-hormonal in origin. In this review, we describe recent basic and clinical findings of the major clinical procedures (adjustable gastric banding, vertical sleeve gastrectomy, Roux-en-Y gastric bypass, and biliopancreatic diversion) as well as other experimental procedures (ileal interposition and bile diversion) that recapitulate many of the metabolic effects of these complex operations in a simpler fashion. As the role of bile acids and the gut microbiome on metabolism is becoming increasingly well described, their potential roles in these improvements following metabolic surgery are becoming better appreciated. Bile acid and gut microbiome changes, in light of recent developments, are discussed in the context of these surgical procedures, as well as their implications for future study.

## Introduction

Obesity is a significant health-care problem with few treatment options, many of which are only minimally effective in the long term. Medical therapy consisting of intensive lifestyle modification (that is, diet, exercise, and behavioral therapy) fails to maintain significant long-term weight loss. Although medical intervention can lead to modest weight loss in select patients ^1^, 5–10% weight loss in a morbidly obese individual still leaves that patient with significant cardiometabolic risk ^2, 3^.

Metabolic and bariatric surgery (in this review, the phrase “metabolic and bariatric surgery” refers to a single entity) is recognized as the most effective treatment for obesity and its associated comorbidities, such as type 2 diabetes ^4– 7^, and its usage continues to increase with the increasing prevalence of obesity and metabolic disease. Early studies from Pories and colleagues ^8^ and others ^9, 10^ in gastric bypass patients described diabetes resolving almost immediately after surgery. Even though this effect was described more than 30 years ago ^8, 11^, its complex underlying mechanisms remain an intense research focus. Multiple reviews and meta-analyses have confirmed a diabetes resolution rate of approximately 80% ^12– 14^ and also provide evidence that operating in patients with a body mass index of less than 35 kg/m ^2^ may be warranted as well for non-obese diabetics ^15– 19^. Overall, the benefits of bariatric and metabolic surgery continue to be better described, particularly the decreases in cardiovascular disease and cancer mortality ^20, 21^. With the alleviation of diabetes and other comorbidities, it is not surprising that bariatric surgery also exhibits cost savings compared with chronic medical treatment of these diseases ^20, 22– 24^.

Our aim herein is not to focus on the anatomic differences of the particular operations per se but instead to highlight the discoveries and new questions each procedure has provided over about the last 5 years. The field of metabolic and bariatric surgery has a rich history, although our understanding of how these operations lead to their beneficial effects has significantly changed over the last 10 to 15 years. Operations that were originally intended to produce weight loss through combinations of gastric restriction or malabsorption (or both) clearly have metabolic benefits that are independent from either one of these previously long-held beliefs of their mechanism of action. The historical bile diversion and ileal interposition operations are scientifically in vogue once again and are helping to examine the complex role of bile acids in metabolic regulation. We have focused on the insights from the most popular procedures clinically and experimentally, including gastric banding, vertical sleeve gastrectomy (VSG), Roux-en-Y gastric bypass (RYGB), biliopancreatic diversion (BPD), ileal interposition, and bile diversion. Importantly, we have emphasized many of the species-specific changes that must be considered when translating findings to a clinical context. The role of bile acids and the gut microbiome and their potential interaction is discussed. For a comprehensive discussion of the rapidly growing field of metabolic and bariatric surgery, we direct the reader to this excellent review ^25^.

## Purely restrictive operations

### Adjustable gastric banding and gastric balloons

**Figure 1.**
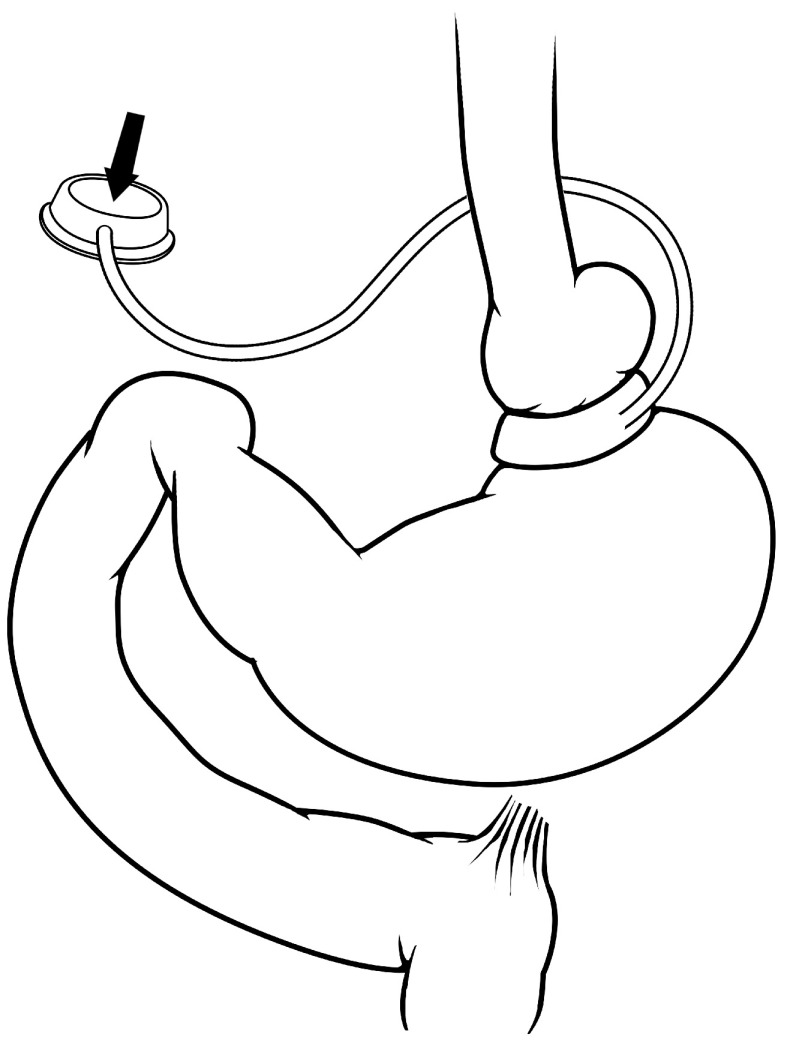
Adjustable gastric banding. In this procedure, an external ring is placed around the proximal portion of the stomach and has a balloon that lines the inside portion of the ring. The inflatable balloon is connected to a port in the subcutaneous tissue of the abdomen that allows the balloon volume, and therefore the amount of gastric restriction, to be adjusted.

The contribution of gastric restriction to the efficacy of bariatric surgery is an area that has been well studied clinically and experimentally. The adjustable gastric band ( Figure 1) and gastric balloons are two procedures that purely decrease the capacity of the stomach, by either an adjustable external compressive device (that is, the adjustable gastric band) or merely taking up space within the stomach (that is, the gastric balloon). Over the last decade, adjustable gastric banding has continued to fall from its peak clinical usage in 2008 to currently comprising only 10% of bariatric procedures worldwide ^26^. This decline is due to the relative ineffectiveness of banding for long-term weight loss and reduced comorbidity compared to other bariatric procedures. Consistent with this trend and clinical findings, gastric banding has been modeled in rodents ^27– 29^ and has been shown to be less effective for long-term weight loss or improvements in glucose tolerance compared with other procedures (for example, RYGB and BPD) that have additional hormonal or malabsorptive characteristics or both greater and more durable effects. In rodent models, gastric banding is purely restrictive and does not confer any additional benefits beyond restriction of food intake ^29– 33^. Banding exerts a temporary weight loss that is compensated for over the course of several weeks ^27^. The delayed resolution of diabetes or other metabolic comorbid conditions is greater with, and attributable to, weight loss secondary to decreased food intake and not direct neuro-hormonal effects ^34, 35^.

Similarly, gastric balloons have recently been approved in the US, although they have been used in Europe for over a decade ^36^. The science behind the balloon is similar to gastric banding in that the balloons are meant to simulate a false sense of gastric distention and promote satiety even after consumption of a minuscule amount of food. Again, similar to the banding procedure, these devices in theory are purely restrictive; however, as they become more popularly used in Europe and the US, further investigation into the potential changes in hormonal or metabolic effects can be studied ^37, 38^.

## More than gastric restriction: operations with hormonal effects

Without a doubt the most exciting advancements in the field of metabolic and bariatric surgery over the last decade have been the identification of mechanisms that have challenged the long-held beliefs that “bariatric” surgical procedures induce weight loss purely through a combination of gastric restriction or nutrient malabsorption, or both. Neural, hormonal, and other nutrient signaling pathways that have previously been unrecognized may be mediating many of the metabolic benefits of these surgical procedures. We examine three of these operations to help highlight these novel and alternative mechanisms in the following section.

### Vertical sleeve gastrectomy

**Figure 2.**
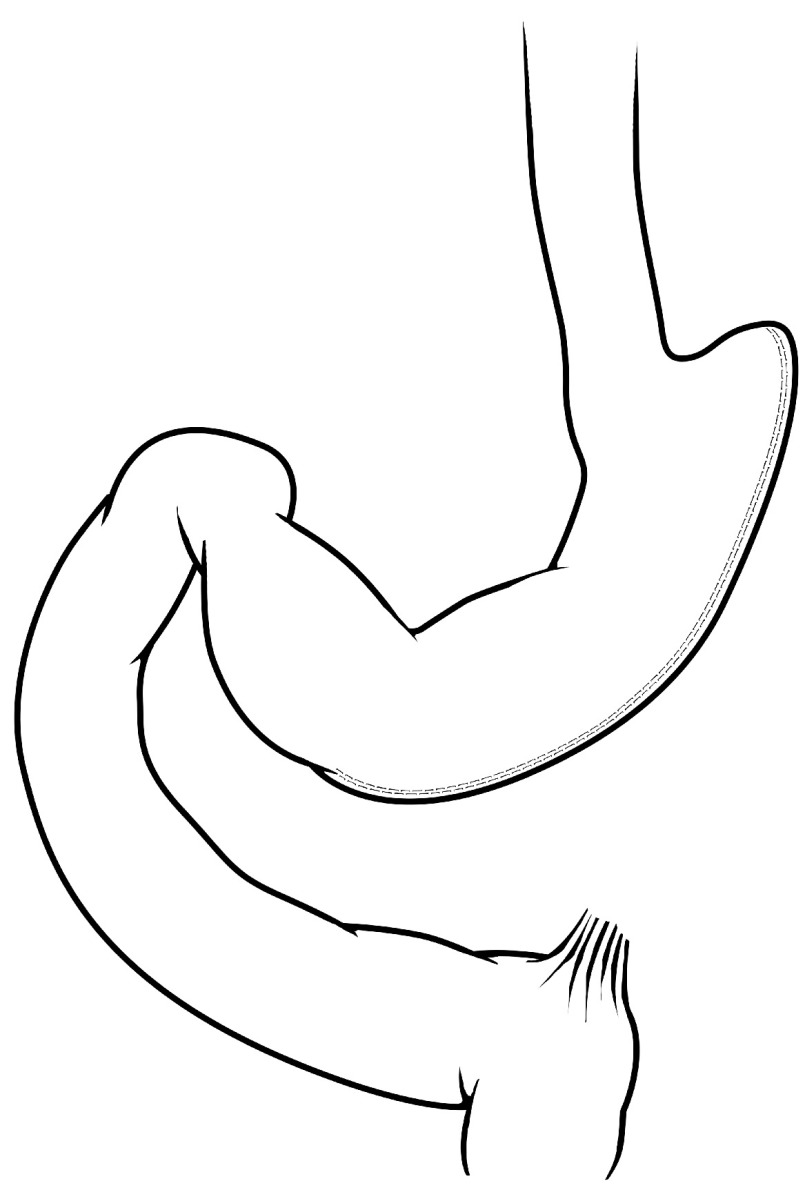
Vertical sleeve gastrectomy. A majority of the greater curvature is excised in this procedure, creating a tube-like stomach with a marked reduction in gastric capacity.

The vertical sleeve gastrectomy is a surgical procedure that decreases gastric volume by approximately 70% with excision of a large portion of stomach along the greater curvature ( Figure 2). As mentioned above, clinical and experimental evidence has demonstrated that gastric restriction alone is not effective as a long-term solution for obesity or its comorbidities. When VSG was first introduced, it was deemed to be a purely restrictive procedure; however, this view has been transformed on the basis of clinical and experimental observations. With better weight loss and metabolic outcomes compared with gastric banding, VSG has increased in usage over the last decade and become almost as popular as RYGB ^26^. However, whether VSG provides similar remission to obesity and diabetes long-term has yet to be determined. The ongoing STAMPEDE trial (Surgical Therapy And Medications Potentially Eradicate Diabetes Efficiently) ^4^, the first prospective randomized clinical trial designed to compare both VSG and RYGB to intensive lifestyle modification alone, is not designed to make direct comparisons between VSG and RYGB. Regardless, the data from the STAMPEDE trial should give us rough insight into how well VSG compares to intensive lifestyle intervention and medical therapy in the long term.

Aside from its clinical usage, VSG has been described in multiple rodent studies and has several interesting physiologic effects that cannot be explained by restriction alone. In fact, data have directly challenged the notion that VSG involves any intrinsic gastric restriction at all. In studies by Grayson and colleagues ^39^, lactating female rats that had previously received a VSG or sham operation were both able to increase their food intake in response to lactation without any evidence of food intake restriction in the VSG rats that were lactating—one of the most energy-demanding processes physiologically when dams routinely double or even triple their food intake ^40^. Initially, VSG was thought to work by excision of the ghrelin-producing portion of the stomach ^41^. Indeed, circulating ghrelin concentrations are significantly decreased up to a year after VSG ^42^. Interestingly, even in the absence of intestinal rearrangement, VSG is associated with increased secretion of the distal intestinal hormones GLP-1 and peptide YY (PYY) ^43– 45^. Studies using rodent models of VSG have examined the contribution of these hormonal changes and other mechanisms to VSG efficacy. Mice with genetic deletion of ghrelin or the GLP-1 receptor continue to show weight loss following VSG ^46, 47^, suggesting that isolated changes in these hormones cannot explain the efficacy of the procedure. Unlike ghrelin and GLP-1 receptor knockout mice, though, mice deficient in the bile acid receptor FXR (farnesoid X receptor) have completely abrogated effects of VSG while on a high-fat diet ^48^, thus implicating FXR as a major target of VSG. Consistent with this bile acid receptor dependency, VSG is associated with increased plasma bile acid concentrations in the mouse ^49^, but whether the same holds true in humans is not year clear ^50^. Stefater and colleagues ^25^ examined the effects of VSG compared with diet-induced obesity, pair-fed, or chow-fed control rats. In that study, total bile acids were increased by VSG or pair-feeding by unknown mechanisms, which began to approach the higher concentrations of total bile acids reported in the chow-fed controls. How FXR signaling is altered by bariatric surgery and what other pathways may be affected by VSG remain unclear, although a number of other VSG-related effects require further study, including changes to taste preference similar to RYGB as well as changes in intestinal triglyceride metabolism ^51– 54^.

### Roux-en-Y gastric bypass and biliopancreatic diversion

**Figure 3.**
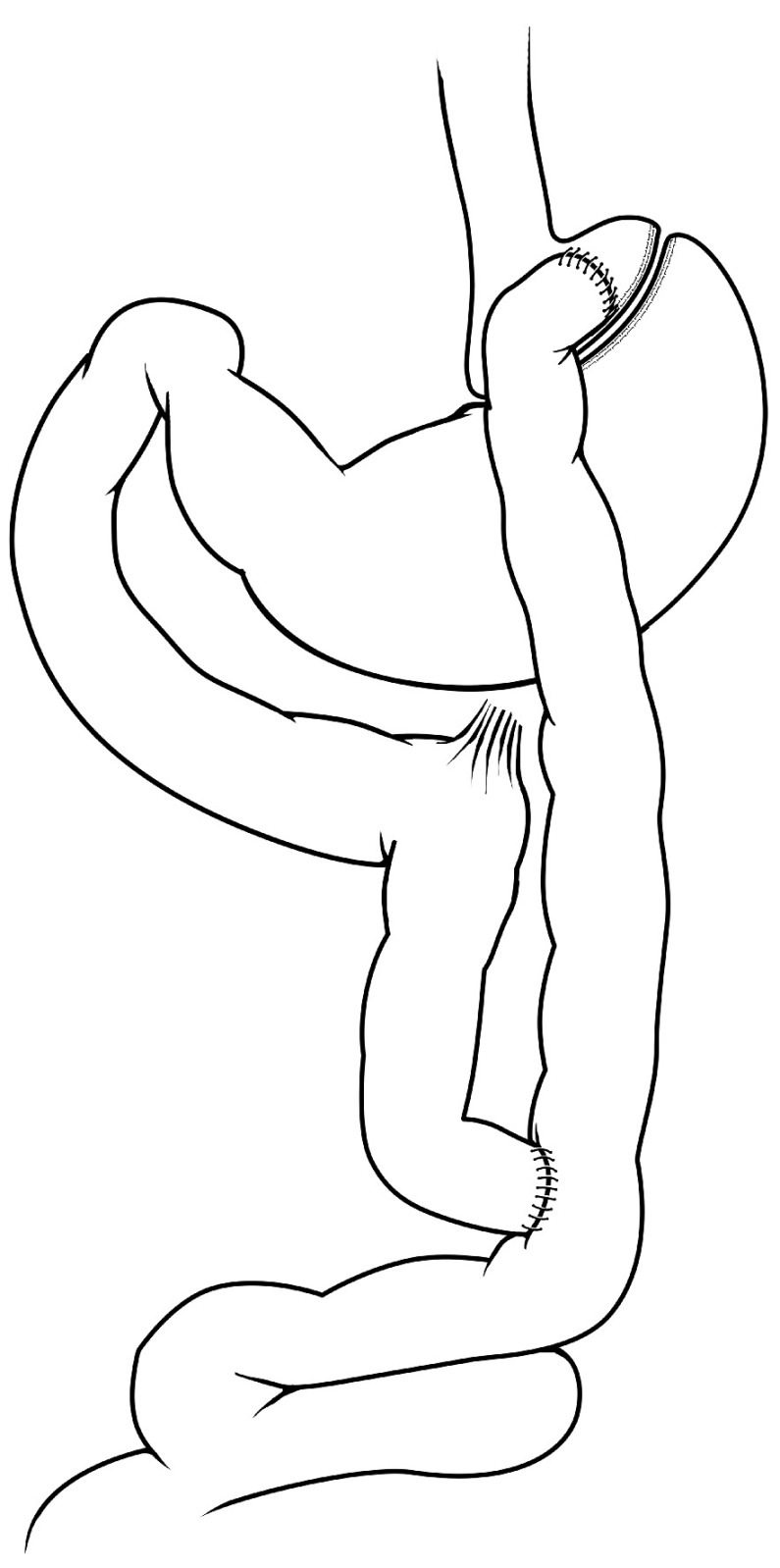
Roux-en-Y gastric bypass. The stomach is divided, creating a small gastric pouch that is connected through a gastro-jejunostomy to a distal segment of jejunum, which forms the Roux limb of the procedure. The remainder of the stomach is referred to as the “gastric remnant” and drains into the bypassed portion of bowel, referred to as the “biliopancreatic limb”. Bowel continuity is restored for the biliopancreatic limb by a jejuno-jejunostomy that creates the “Y” configuration of the operation. Thus, ingested nutrients proceed rapidly through the stomach pouch and move immediately into the jejunal Roux limb in the absence of bile and pancreatic secretions. Bile and pancreatic secretions drain via the biliopancreatic limb and then mix with the chyme/nutrients at the point of the jejuno-jejunostomy.

**Figure 4.**
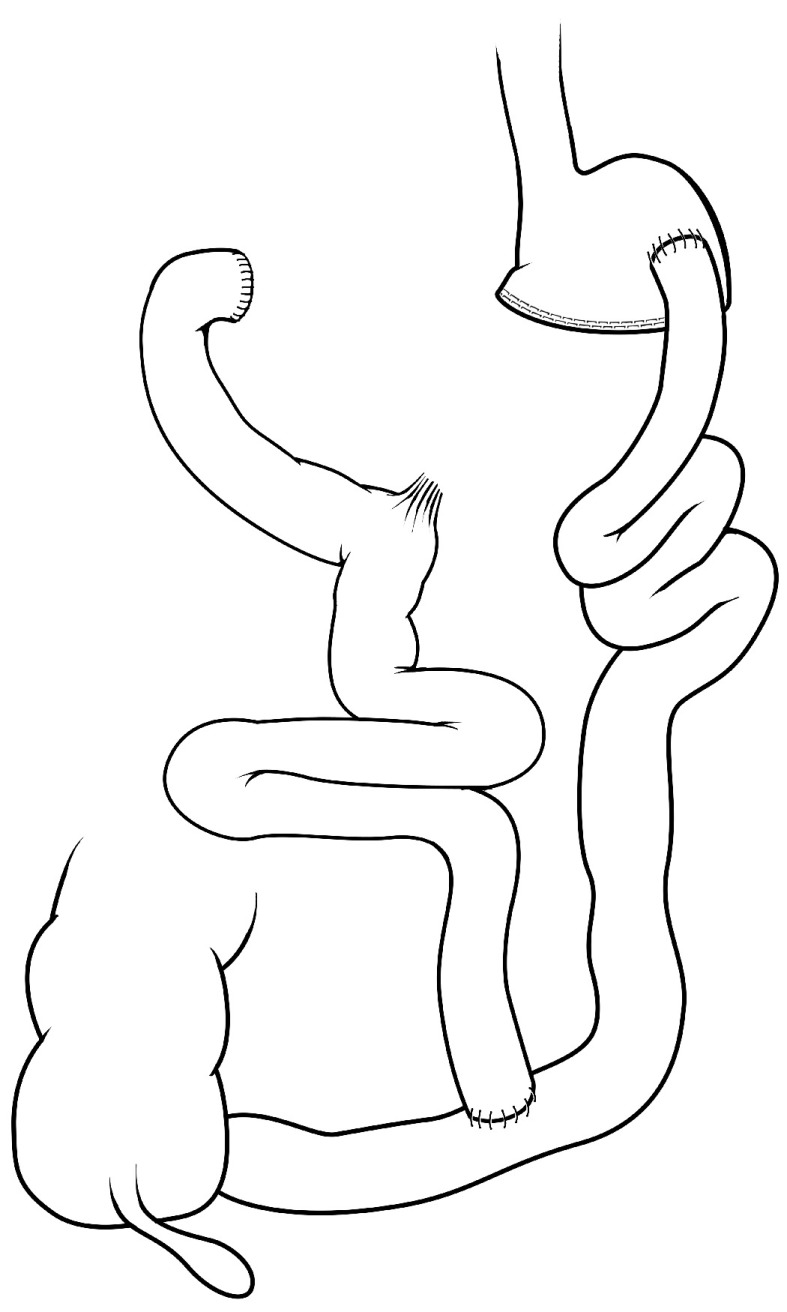
Biliopancreatic diversion. This is a procedure that effectively diverts bile and pancreatic secretions to the distal bowel for mixing with nutrients/chyme, typically much further distal than a Roux-en-Y gastric bypass. This procedure can be performed with or without a partial gastrectomy and is also referred to as a duodenal switch; the “switch” is the diversion of bile and pancreatic secretions from nutrient flow.

The RYGB ( Figure 3) and BPD ( Figure 4) operations combine significant intestinal rearrangement with gastric restriction. Each procedure involves creation of a smaller stomach pouch while diverting nutrient flow to varying distal segments of the intestine. The gastric restrictive component is typically less with BPD but diversion of biliopancreatic secretions is more distal, compared with RYGB. Both RYGB and BPD were originally thought to cause weight loss through a combination of malabsorption and gastric restriction. From clinical practice, we know that bariatric surgical patients are at higher risk for certain micronutrient deficiencies, highly suggestive of an intrinsic malabsorptive component leading to weight loss after these procedures ^55– 57^. Compared with RYGB patients, BPD patients tend to have more nutritional and gastrointestinal (GI) side effects ^58^, which may indicate a more severe malabsorptive phenotype. Regardless, both operations produce improvements in diabetes/insulin resistance ^59– 62^. Several studies have attempted to determine the magnitude of malabsorption following these procedures clinically and experimentally. In terms of macronutrient malabsorption, animal studies suggest a higher degree of malabsorption in RYGB/BPD models, which could contribute to significant weight loss ^63, 64^. However, this degree of malabsorption is not consistently observed in practice, and several clinical studies show frank macronutrient malabsorption, minimal macronutrient malabsorption, or no macronutrient malabsorption at all ^65– 68^. Both nutrient and macronutrient malabsorption appear to be much more easily observed in the BPD models compared with RYGB ^65, 69^. Collectively, macronutrient malabsorption appears to play a much greater role in the case of BPD than RYGB in the weight loss observed in these patients clinically. However, the complex intestinal rearrangement, altered nutrient absorption, and physical separation of biliopancreatic secretions from nutrients in these operations may alter the intestinal nutrient milieu to explain what drives many of the hormonal and histologic changes ^70^ observed in these procedures.

Given the anatomic changes with RYGB and BPD, two opposing hypotheses—referred to as the “foregut hypothesis” and the “hindgut hypothesis”—arose in the field. The foregut hypothesis posits that an unknown factor—neural or hormonal or both—originates from the bypassed intestinal region in RYGB/BPD that promotes insulin resistance. Thus, bypass of that segment is associated with metabolic improvements. There is considerable evidence in support of this hypothesis, proposed as early as the 1980s by Scopinaro and colleagues ^71^, although the identity of such a factor remains elusive ^72, 73^. Two studies examined the foregut hypothesis directly in RYGB patients with gastrostomy tubes in the gastric remnant ^74, 75^. Hansen and colleagues ^74^ found that reintroduction of nutrients into the excluded foregut in the first 6 weeks after RYGB did not reverse improvement in glucose responses, whereas Pournaras and colleagues ^75^ found reversal between 9 and 24 months after RYGB. Differences between these two studies could be related to early-versus-late post-operative effects or possible enteral feeding prior to testing in the latter study. Animal studies have also had mixed results, showing that duodenal nutrient exclusion is associated with villous hyperplasia but that improvements in glucose tolerance, weight loss, or incretins are mixed and may depend heavily on strain/genetic background ^76– 79^. Conversely, the “hindgut hypothesis” posits that the more rapid delivery of nutrients to the hindgut stimulates either neural or hormonal factors (or both) that lead to the metabolic benefits and weight loss. Within the first month after surgery, concomitant increases in postprandial GLP-1 and insulin secretion and an enhanced incretin effect are observed and have positioned GLP-1 as the prime mediator of improved glucose homeostasis after the procedure. Recent data using GLP-1 receptor antagonists have challenged a dominant role for GLP-1. Although these data indicate that the increased GLP-1 contributes to the increased insulin secretion, this does not translate into improved glucose homeostasis ^80^. It is somewhat unfortunate that these opposing theories arose, because the field has come to appreciate that there are likely components of each that could potentially be at work following RYGB and BPD.

Aside from foregut bypass, a number of anatomic/histologic changes are associated with the intestinal rearrangement that can be potentially linked to the metabolic changes. Cell proliferation and villous surface area are increased in the Roux limb of RYGB in humans, as are expression levels of genes suggestive of increased energy demands ^81^. A working hypothesis in rats is that Roux limb hypertrophy, secondary to energetic demands on the jejunum, results in increased nutrient uptake following RYGB, making the Roux limb a significant contributor to glucose homeostasis. This is evident by increased basolateral glucose uptake of the Roux limb in the post-absorptive state with corresponding changes in glucose transporters on immunohistologic analysis ^82^. Consistent with this intestinal hypertrophy hypothesis, other procedures have also shown changes in intestinal histology and increased L-cell populations with concomitant changes in L-cell hormonal responses (that is, GLP-1 and PYY) ^64, 83, 84^. However, histologic changes also occur in the common channel (that is, distal ileum) that could represent hypertrophy secondary to undigested luminal nutrients ^85^.

Besides the improvements in insulin sensitivity/glucose homeostasis and weight loss observed with RYGB, nutrient signaling within the GI tract to alter taste or other metabolic processes is significantly changed by these operations. Several studies have demonstrated altered taste preference for lipid or glucose solutions in humans and animals after RYGB ^52, 86– 88^. These findings appear to reflect changes in central reward pathways, but whether this reflects direct central effects or actions of peripheral signals needs to be determined ^88– 91^. Additionally, similar neural sensing mechanisms operating via the vagus nerve have been implicated in luminal nutrient sensing, which adds another layer of complexity between the neural and hormonal regulation that is changed by RYGB and potentially other bariatric operations. Further studies are needed to identify these mechanisms, as the role of the vagus nerve in these effects remains unclear ^92– 94^.

Although most of the focus has been placed on the intestines, considerable evidence indicates that negative energy balance may also have beneficial metabolic effects after RYGB. In the immediate post-operative period, significant caloric restriction contributes to the early improvements in glucose metabolism ^95– 97^. Within the first month after surgery, improvements in hepatic insulin sensitivity are evident ^98, 99^, indicating an important contribution of the liver in mediating the weight loss-independent effects of RYGB. On the other hand, an improvement in peripheral insulin sensitivity, which mediates glucose disposal after a meal, occurs later and is related to the ensuing weight loss ^99– 101^. It has been debated whether an increase in energy expenditure is responsible for weight loss post-operatively in these patients. Recent evidence suggests that resting energy expenditure is increased in mice ^63, 102^ as well as rats ^103, 104^ following RYGB. Unlike animal data, human studies using appropriate methodology (that is, regression modeling ^105– 107^) do not support any increases in energy expenditure following RYGB ^108, 109^. In fact, the massive weight loss of RYGB occurs in the setting of metabolic adaptation (decreased energy demands greater than expected for the degree of weight loss), suggesting the contribution of hormonal or neuronal factors (or both) in the anorectic effect ^110^. The effects of RYGB on energy expenditure appear to be contrary to those observed in BPD, which Scopinaro and colleagues have reported ^111^. Overall, when examining the metabolic and body weight changes observed both acutely and chronically by RYGB and comparing those with BPD, researchers are tempted to speculate that the BPD operation represents an extreme physiologic response. A response of this magnitude tends to make sense given the apparent effects of BPD on energy expenditure, malabsorption, and long-term body weight, which are more easily observed with that procedure compared with RYGB.

## Experimental metabolic operations without gastric restriction

### Ileal interposition

**Figure 5.**
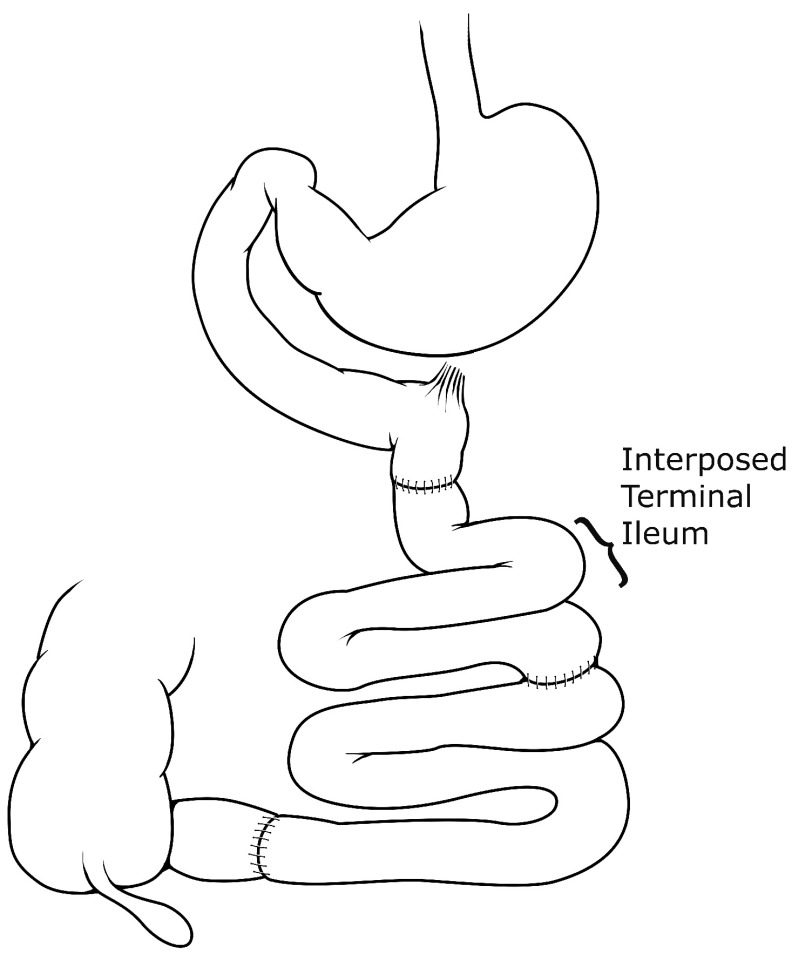
Ileal interposition. A neurovascular intact segment of distal or near-terminal ileum is interposed in the proximal jejunum near the ligament of Treitz. The distal jejunum is then re-anastomosed to the small segment of ileum proximal to the ileocecal valve to re-establish bowel continuity.

The ileal interposition ( Figure 5), also previously referred to as “ileal transposition”, has been an insightful procedure used to examine the mechanisms underlying altered nutrient flow after RYGB or BPD. The procedure involves taking a neurovascular intact segment of near-terminal ileum and interposing it just distal to the ligament of Treitz, effectively producing a model simulating rapid hindgut delivery like RYGB or BPD, but without any gastric restriction. Koopmans and colleagues ^112^ first identified that ileal interposition surprisingly led to decreased food intake in obese rats, which the authors attributed to an “over-stimulated ileum”. In most cases, however, these changes in food intake or body weight are either negligible or modest and this may explain why they are not uniformly detectable—indicating potential importance of genetic background (that is, animal strain) or feeding/housing methods in rodent studies ^113– 116^. Moreover, these changes appear to be heavily dependent on the length of the ileal segment interposed; longer interposed segments have more robust metabolic effects that could account for variability of findings ^117^. Consistent with this over-stimulated ileal hypothesis, the ileal interposition has profound effects on glucose homeostasis as well as GI hormone secretion and bile acid metabolism similar to RYGB, BPD, and VSG. Weight-independent improvements in glucose homeostasis in rats are secondary to improved skeletal muscle glucose uptake and insulin signaling via Akt ^113^. These benefits persist in monogenic and polygenic rat models of obesity or diabetes, or both ^113, 114^. The interposition procedure is also associated with increased expression and secretion of ileal hormones (that is, GLP-1 and GIP) ^113, 115, 116^ that correspond to increased enteroendocrine cell numbers ^118^.

Aside from ileal hormones, ileal interposition causes marked elevations in circulating bile acids ^113, 116^, the mechanism of which remains unclear. However, both basal and nutrient-stimulated bile acid concentrations are a common finding after bariatric operations. In this regard, bile acids are recognized players in the regulation of glucose and lipid metabolism through the FXR and TGR5 receptors, respectively. It is tempting to speculate that activation of those receptors could improve glucose tolerance and enhance insulin sensitivity.

The mechanisms responsible for the observed elevations in plasma bile acid concentrations following ileal interposition remain unclear. Strader and colleagues ^119^ suggested that the beneficial effects of ileal interposition come from nutrient or bile exposure of the interposed ileal segment at much higher concentrations, thus overwhelming the transposed segment and subsequently causing compensatory changes to the “neo-ileum”. In rats, following ileal interposition the apical sodium bile acid transporter is decreased (~95%) in the interposed ileal segment, although the cytosolic transporter is increased. In those same animals, however, the most distal segments of intestine (that is, the remaining or neo-ileum and/or colon) had robust increases in expression of these transporters ^119^.

### Bile diversion

**Figure 6.**
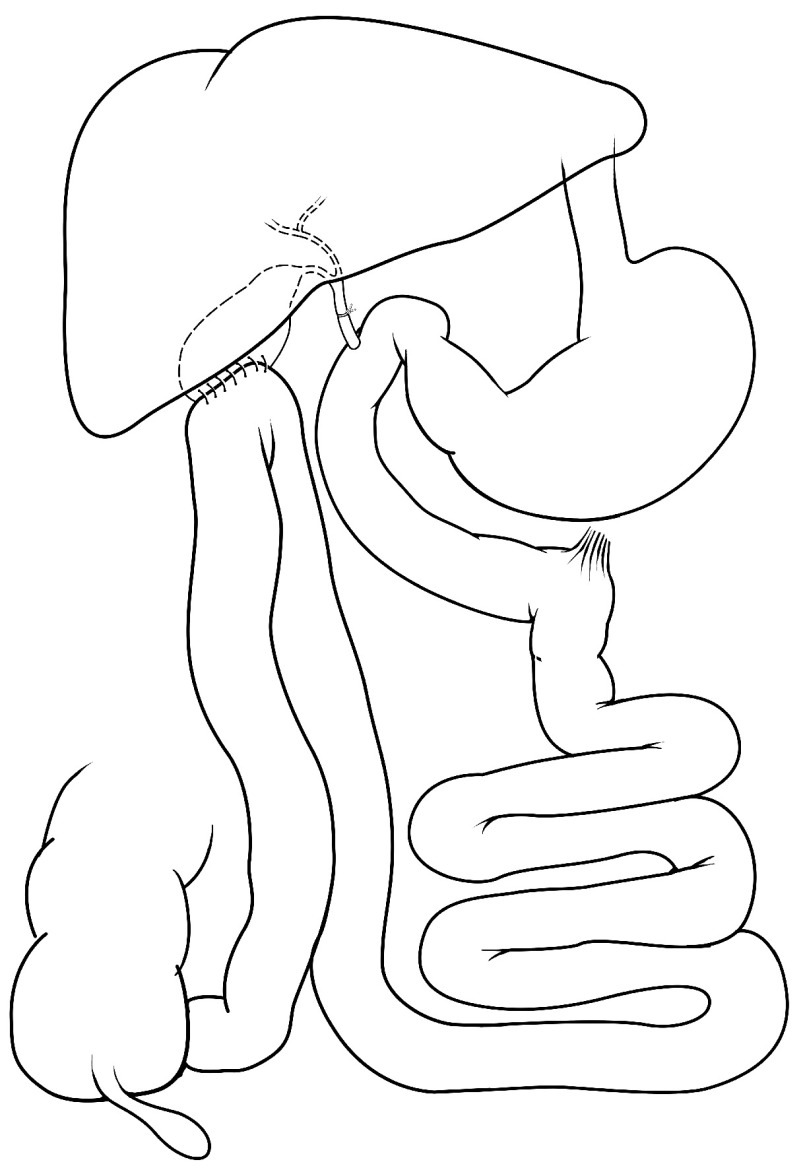
Bile diversion. In the absence of any gastric restriction, the common bile duct is ligated proximal to the pancreatic duct and an anastomosis is created between a portion of ileum and the gallbladder. Pancreatic secretions follow their normal course and drain into the duodenum, but biliary secretions are diverted to the portion of ileum connected directly to the gallbladder.

Bile diversion ( Figure 6) was an experimental surgical technique developed in the 1960s ^120, 121^ for surgical management of hypercholesterolemia. The proposed mechanism of the procedure was to prevent bile salts from mixing with intestinal contents prior to reaching the colon, and thus cholesterol (and other lipid) absorption would be significantly less. The bile diversion procedure paralleled the development of the jejunal-ileal and ileal bypasses pioneered by Buchwald and Gebhard ^122^ at the University of Minnesota and Scott and colleagues ^123^ at Vanderbilt University, and this culminated in the Program of Surgical Control of Hyperlipidemias (POSCH) trial ^124^. From these studies, as one would expect, there were tremendous improvements in total cholesterol levels that were attributed to the lack of bile-nutrient mixing and thus retarded lipid absorption.

At the time bile diversion was developed, bile acids had not yet been recognized as the metabolic hormones that we know today ^125, 126^. Thus, more recent studies have revitalized the potential uses for bile diversion as a model to dissect the mechanisms at work following bariatric surgery because of its similarities to other bariatric procedures. Bile diversion, similar to RYGB or BPD, limits bile-nutrient mixing until a more distal point in the intestine. Unlike the RYGB or BPD, though, there is no biliopancreatic limb for these secretions to flow; they are completely diverted by cannula or anastomosis directly to the intestinal segment of interest maintaining unaltered alimentary flow. Coincidentally, it was noted that glucose tolerance was improved even in the absence of weight loss in dogs after bile diversion ^127^. Manfredini and colleagues ^128^ replicated these observations and described improvements in oral and intravenous glucose tolerance in the absence of any changes in insulin secretion. However, a recent report of bile diversion in lean rats has shown conflicting results on whether insulin secretion is altered, and this requires further study ^128, 129^.

Regardless of whether insulin secretion is altered, there is improved insulin responsiveness and decreased fasting glucose after bile diversion suggestive of a change in insulin sensitivity in lean rats ^129^. Similarly, in a high fat-fed model, Kohli and colleagues ^130^ have shown that bile diversion to the jejunum produces weight loss compared with sham/control rats. These beneficial changes are recapitulated with oral bile acid administration and appear to be mediated by alleviating endoplasmic reticulum stress. Moreover, bile diversion in rats was associated with increased number and length of villi, similar to procedures like RYGB, BPD, and ileal interposition ^130, 131^. Each of the bile diversion models is associated with increased circulating bile acid concentrations, which appear to be necessary for the metabolic effects since administration of bile acid sequestrants (for example, cholestyramine) normalizes plasma bile acid concentrations and abolishes the metabolic improvements. These findings are consistent with our recent studies using a gallbladder to ileum anastomosis in the mouse, which compared it alongside RYGB in a mouse model of diet-induced obesity. Similar to the findings in rats, bile diversion in mice is associated with striking improvements in glucose tolerance, insulin sensitivity, normalization of blood lipids, and complete resolution of hepatic steatosis ^63^. These changes are secondary to a degree of malabsorption of dietary lipid in the mice; however, they are also observed with marked increases in total circulating bile acids—specific bile acids that are implicated in metabolic signaling through bile acids receptors (that is, TGR5 and FXR) ^125, 132^.

## Role of bile acids and the gut microbiome

The contributions of bile acids in the metabolic effects following bariatric surgery are at the forefront of current investigation. There is considerable evidence that bile acid concentrations are increased following bariatric surgical procedures—both clinical and experimental—and the most robust clinical changes are observed in RYGB and BPD ^133– 138^. Whether the changes in circulating bile acid concentrations lead to changes in known downstream metabolic effectors like fibroblast growth factor 19 (FGF19, FGF15 in rodents) is still under investigation ^139^. The changes in bile acid metabolism and circulating concentrations appear to be dynamic following RYGB ^133^ and this may explain why some investigators observe increases in the metabolically beneficial hormone FGF19 and some do not ^133, 138, 140– 142^. From a mechanistic perspective, the fact that the ileal interposition and bile diversion procedures recapitulate these bile acid elevations is quite intriguing and implies that either absence of bile in the proximal intestine or overabundance in the distal intestine may contribute the metabolic effects observed. The changes in bile acids appear to be mediated in part by increases in bile acid synthesis through increased expression of CYP7A1, the rate-limiting enzyme in bile acid synthesis. However, there are also concurrent changes in bile acid transporter proteins in the liver and the ileum ^63^ that would also be expected to increase the circulating total bile acid pool. Paradoxically, bile acid synthesis is elevated in the face of increased SHP and decreased FXR expression in the livers of these animals. These changes in SHP and FXR expression are inconsistent with our current understanding of hepatic bile acid synthesis ^143^. Regardless of how bile acid concentrations are increased following the ileal interposition and bile diversion procedures, the molecular mechanisms of how the increased abundance of bile acids may alter glucose homeostasis is currently unknown. Evidence suggests that these effects are mediated via FXR or TGR5 signaling (or both) in the beta-cell or in the enterocytes themselves (or in both) ^125, 144, 145^, but further studies need to focus on clarifying these mechanisms and their physiologic importance. These bile acid-mediated effects on glucose and lipid metabolism have therapeutic implications on diabetes irrespectively of changes on body weight and are a focus of current investigation.

Perhaps one of the most intriguing observations of this century thus far has been that fecal transfer of gut microbiota from obese donors causes weight gain in lean recipients ^146^. Similarly, the opposite effect can be demonstrated in obese mice with stool from RYGB mouse donors, which demonstrates the transferability of at least some of the metabolic benefits of gastric bypass ^147^. There has been significant evidence that the gut microbiota is altered following bariatric surgery, although the mechanisms of these changes and the potential contribution they make in the metabolic benefits post-operatively are unknown. Changes in the microbiota have been examined in mouse models of sleeve gastrectomy ^48^ as well as biliary diversion ^63^, and these procedures show what appear to be beneficial changes. Interestingly, le Roux and colleagues ^148^ have recently shown that the gut microbiota alter the pattern of adipose tissue deposition. The interaction between the gut microbiota and bile acid metabolism is complex, and particular bile acids are antibacterial and likely affect the gut microbiota. In contrast, the gut microbiota are the major source of bile acid diversity, chemically transforming endogenously produced bile acids to a number of different chemical species that likely have varying potencies at bile acid receptors. At this time, it is not clear whether these changes in microbiota are a cause or an effect of the metabolic improvements and weight loss observed following bariatric surgery, but the potential for altering the microbiome as a treatment for obesity or diabetes (or both) continues to emerge ^149^.

## Summary and future directions

From a clinical perspective, prospective clinical trials are needed to directly compare not only durability of weight loss but also resolution of other obesity comorbidities (for example, diabetes, insulin resistance, and hyperlipidemias) after VSG and RYGB ^150^. Scientifically, while much progress has been made regarding the weight loss-independent metabolic effects of bariatric surgery, the complexity of the system continues to provide challenges. The field is progressing from a “GLP-1-centric view” of bariatric surgery to encompass the importance of early benefits of caloric restriction and non-traditional regulators of metabolism. The changes in bile acid metabolism with bariatric surgery will continue to drive the study of these procedures and the mechanisms in animal models—especially as the bile acid field continues to advance our understanding of these potent metabolic regulators. Lastly, the interaction of the bile acid milieu and the gut microbiome cannot be ignored. The gut microbiome field continues to grow and become more intervention-driven, as opposed to descriptive, and will continue to help identify the changes underlying the metabolic benefits of bariatric and metabolic surgery. These continue to be exciting times for metabolic and bariatric surgical research, and future studies examining the contributions of bile acids and microbiota both clinically and experimentally may lead to more effective treatments, and perhaps new interventional procedures for obesity and diabetes.
